# INDOOR AND REAL-LIFE OUTDOOR GAIT SPEED AND ITS ASSOCIATION ON GEOGRAPHIC LIFE-SPACE AND OUTDOOR MOBILITY

**DOI:** 10.1093/geroni/igz038.1761

**Published:** 2019-11-08

**Authors:** Chek Hooi Wong, Pey June Tan, Mei Sian Chong, Jagadish U Mallya, Mimaika L Ginting, Isabel H Ng, Soon Hoe Ho, Belinda Yuen

**Affiliations:** 1 Geriatric Education and Research Institute, Singapore, Singapore; 2 Department of Geriatric Medicine, Khoo Teck Puat Hospital, Singapore, Singapore; 3 Singapore University of Technology and Design, Singapore, Singapore

## Abstract

Independent outdoor mobility is important to community-dwelling older adults as it enables reach and access to resources for everyday activities, but this becomes increasingly challenging with the progressive decline in physical performance in ageing. We aim to understand the relationship between Indoor (IGS) and Real-Life Outdoor Gait Speed (OGS) with objectively-measured geographic life-space extent and outdoor mobility among community-ambulant older adults in Singapore. Thirty-three participants aged ≥55 years living in three neighbourhoods wore hybrid mobility trackers continuously for 7 days. Baseline 6-metre IGS was measured with a stopwatch, while OGS was from outdoor accelerometer data. Nodes were defined as significant places visited for ≥5 minutes. Multiple linear regressions examined each association between IGS and OGS on geographic life-space extent and outdoor mobility measures adjusting for confounders. Participants’ mean age was 69.2±7.1 years with mean IGS and OGS of 1.11m/s and 0.85m/s respectively. They spent on average 4.7 hours/day out-of-home with the majority (57%) of nodes located within 500m from home. There were no significant associations between gait speeds and geographic life-space measures. Higher OGS was associated with higher total number of nodes/week while higher IGS was significantly associated with lower percentage of nodes within 500m from home. Our findings highlighted the complexity and multi-dimensionality of independent outdoor mobility as both gait speed performances were not significantly associated with geographic life-space extent, but rather with spatiotemporal interaction and opportunities to access locations for older adults’ daily activities.

